# Simulated and Real Sheet-of-Light 3D Object Scanning Using a-Si:H Thin Film PSD Arrays

**DOI:** 10.3390/s151229779

**Published:** 2015-11-30

**Authors:** Javier Contreras, Josep Tornero, Isabel Ferreira, Rodrigo Martins, Luis Gomes, Elvira Fortunato

**Affiliations:** 1 Institute of Design and Manufacturing, Technical University of Valencia, CPI, Edif. 8G, 46022 Valencia, Spain; jtornero@idf.upv.es; 2 CENIMAT/I3N, Department of Material Science, Faculty of Science and Technology, FCT, New University of Lisbon and CEMOP/UNINOVA, 2829-516 Caparica, Portugal; imf@fct.unl.pt (I.F.); emf@fct.unl.pt (E.F.); 3 Department of Electrical Engineering, Faculty of Science and Technology, FCT, New University of Lisbon, and CTS/UNINOVA, Campus da Caparica, 2928-516 Caparica, Portugal; lugo@uninova.pt

**Keywords:** three-dimensional sensing, arrays, three-dimensional image acquisition, optical sensing and sensors, thin film devices and applications, three-dimensional image processing

## Abstract

A MATLAB/SIMULINK software simulation model (structure and component blocks) has been constructed in order to view and analyze the potential of the PSD (Position Sensitive Detector) array concept technology before it is further expanded or developed. This simulation allows changing most of its parameters, such as the number of elements in the PSD array, the direction of vision, the viewing/scanning angle, the object rotation, translation, sample/scan/simulation time, *etc*. In addition, results show for the first time the possibility of scanning an object in 3D when using an a-Si:H thin film 128 PSD array sensor and hardware/software system. Moreover, this sensor technology is able to perform these scans and render 3D objects at high speeds and high resolutions when using a sheet-of-light laser within a triangulation platform. As shown by the simulation, a substantial enhancement in 3D object profile image quality and realism can be achieved by increasing the number of elements of the PSD array sensor as well as by achieving an optimal position response from the sensor since clearly the definition of the 3D object profile depends on the correct and accurate position response of each detector as well as on the size of the PSD array.

## 1. Introduction

Sheet-of-light range imaging is an interesting technique that is used in a number of 3D object rendering applications. Among the existing laser scanning techniques, a structured light triangulation method is considered to be fastest when acquiring 3D data information from an object in real time [1]. Generally, sheet-of-light systems use digital sensors such as CCDs (Charged Coupled Devices) or CMOS (Complementary Metal Oxide Semiconductors) [2], nevertheless, analog sensors such as arrays of PSDs (position sensitive detectors) with reported sizes of up to a maximum of 128 have also been employed [3,4]. The latter are fabricated using crystalline silicon, however, PSD arrays based on amorphous silicon nip or pin structures also exist and have already been described elsewhere [5,6]. In this work, results show for the first time the possibility of scanning an object in 3D when using an a-Si:H thin film 128 PSD array system and, in addition, a MATLAB/SIMULINK software simulation has been constructed in order to view and analyze the potential of PSD array concept technology before it is further expanded or developed. This study will enable the triangulation platform and system to be further developed and enhanced for future needs identified during simulations. Inspired by the simulation, examples of improvements and future adaptations could be the inclusion of suitable 360°rotation plates, faster translation tables, more precise optics and laser lines as well as larger sensor arrays depending of course on the limitations imposed by such a scenario. This simulation allows changing most of its parameters, such as the number of elements in the PSD array. The existing 128 PSD array sensor system, when mounted in a triangulation platform, is able to perform 3D object profile scans at high resolutions and speeds with a large number of frames, and this experiment was already presented here. Our previous works describe the implementation, characteristics and behavior of such 32/128 PSD array sensor systems, as well as the manner in which the scanned 3D object profile image is constructed thereby presenting a high speed sheet-of-light 3D object rendering platform [7] using as sensor an array of 32 amorphous silicon position sensitive detectors [6]. In the most recent work [8], we also exploited the 3D scanning optical characteristics of such an inspection system. 

### Analog versus Digital Technology

The foreseen PSD array technology roadmap proposes the use of amorphous silicon or other more suitable materials such as nanocrystalline silicon (which does not degrade as much), for the fabrication of analog 32/128/256/512/1024 PSD array sensors. The latter are used to scan and represent 3D profiles of objects in real-time [8], preferably at a greater speed than digital CCD or CMOS-based systems.

AT-Automation Technology GmbH [9], manufactures the most advanced CMOS sensor based sheet-of-light laser triangulation 3D cameras. They use digital sensors such as CMOS sensors (e.g., 1280 × 1024 pixels). The principle of application for these 3D sheet-of-light cameras is exactly the same as the one used for the 3D PSD sensor system and thereby they are the most advanced direct competitors in terms of speed, resolution and overall system performance, since they claim their cameras are the fastest in the world. One of their cameras (C4-1280) reaches 40,000 profiles/frames per second, when using 128 pixels and 128 rows of the sensors, representing only just a small part of the whole sensor (1280 × 1024 pixels).

Amorphous or nanocrystalline 32/128/256/512/1024 PSD array 3D sensors would be an alternative to these CMOS cameras in similar application scenarios. The overall performance between both technologies will be similar when using the 128 PSD sensor, however, amorphous or nanocrystalline 256/512/1024 PSD array 3D sensors are expected to outperform these 3D CMOS cameras when used in high speed 3D scanning applications. The reason for this is that the analog structure of the PSD sensor is different to the traditional discrete sensor and thereby it can process data much faster than a pixel-based structure. The resolution of these 3D CMOS cameras is quite good, depending on the application, and, in some applications, they claim to reach resolutions of about 35 µm or even 10 or 5 µm [9].

Therefore, it seems 3D cameras with digital sensors are already quite advanced and relatively cheap. A complete set generally includes a camera, laser and lens but not the software; nevertheless, third party software is readily available. 

## 2. 128 PSD Array System 3D Object Profile Scanning

**Figure 1:**
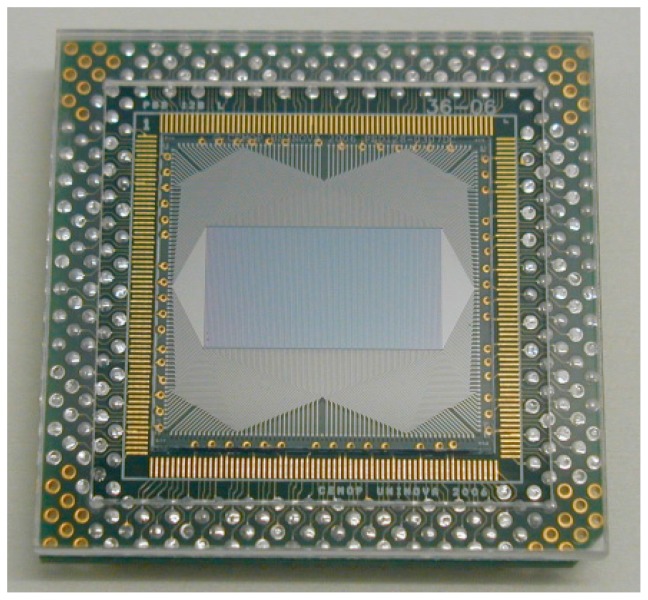
Amorphous silicon 128 PSD array glued and wire bonded to a suitable chip carrier.

As already described in our previous work [7], an array of 32 amorphous silicon position sensitive detectors, was integrated inside a self-constructed machine vision system as the vision sensor component and the response was analyzed for the required application. Such work proposed the use of these sensors and relevant systems for 3D object profiling even at high speeds. A photograph of an amorphous silicon 128 element PSD linear array developed at CEMOP/UNINOVA is shown in Figure 1.

This device can also be regarded as a three-dimensional PSD, being simply an array of one-dimensional PSDs mounted together in parallel to each other on a surface, where the separation between them and the detector width defines the minimum discrimination in one direction (discrete), while in the other (along the strip) it is continuous [6]. This type of structure has been specially designed for sheet-of-light 3D shape measurement.

**Figure 2:**
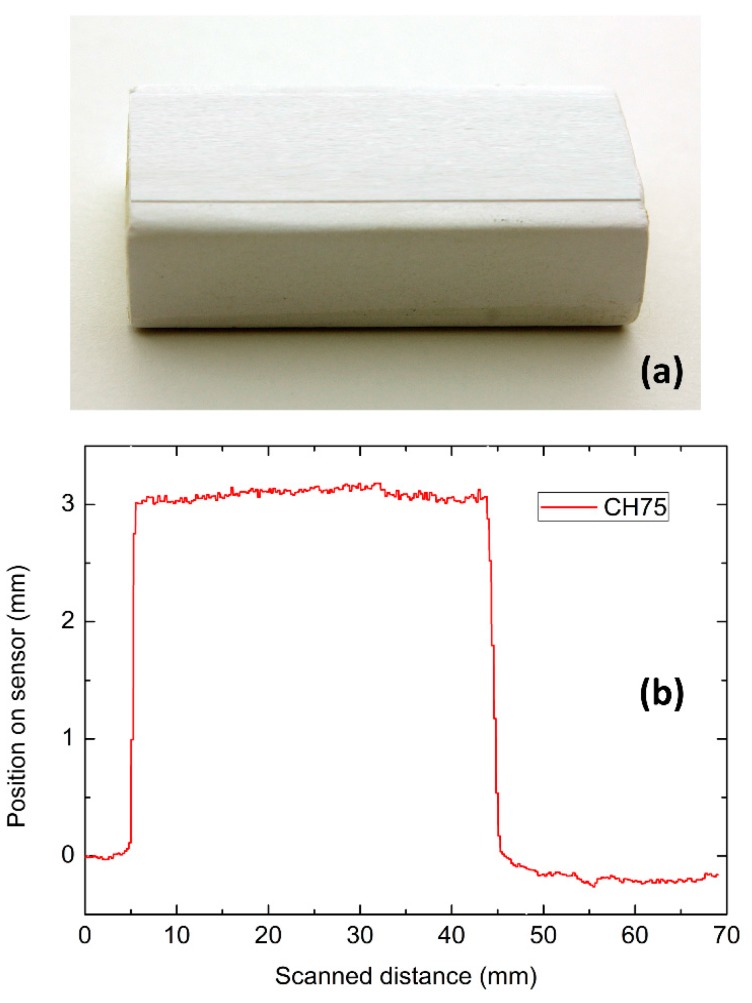
(**a**) Photograph of a white rubber object; (**b**) 3D object profile representation for the individual detection of channel 75 from the 128 PSD array sensor.

The experimental setup and procedure for obtaining 3D object profiles with the 32 PSD sensor system were already described elsewhere [7,8] and these also apply for the 128 PSD sensor system. Here, we show results for the detection of the same object when using the 128 PSD array sensor system. The width of the rubber is small and it is only covered or detected by a few sensor channels. In addition, in these trials, the translation table (and object) moved from one side to the other so that the object (white color rubber), shown in Figure 2a, was scanned by the laser sheet-of-light system.

The object reflection was projected onto the active area of the 128 PSD sensor array. The dimensions of the rubber object, as measured by an electronic ruler, were the following: Length, 41.90 mm; Width, 16.70 mm; and Height, 11.74 mm.

The total distance travelled by the translation table (object) or scanned distance was about 69.37 mm at a speed of about 0.197 cm/s. As already reported elsewhere [8], here the incident scanning angle was fixed at 45° and the rate of acquisition was kept at 8 ms. The system integration time was maintained at 1ms for all scans and for low noise purposes, results were acquired using 128 sub-samplings (128 sample average). The light intensity recorded on the active area of the 128 PSD sensor array was 2.53 µW/cm².

The results obtained when channel 75 of a 128 PSD sensor array detects the light reflected by the white rubber as a function of the scanned distance are illustrated in Figure 2b. The response was acquired at 65 frames per second leading to 2289 frames in total.

The maximum resolution that can be obtained with an acquisition time of 8 ms derives from the number of maximum possible frames acquired in one second taking into account the lowest possible scanning speed. Therefore, 1/0.008 s = 125 frames per second is the maximum possible acquisition frame rate at present (WINDOWS limitation) for this particular system configuration. Using the previously referred to acquisition time of 8ms and speed of 0.197 cm/s, a calculated scanning resolution of around 15.76 μm should be expected (0.00197/125 = 15.76 μm). However, in reality it takes more than 30 μm to acquire each frame, mainly due to software execution internal timings, *etc*.

The response shown in Figure 2b corresponds fairly well to the measured length of the rubber object being 41.90 mm as measured by an electronic ruler. Channel 75 of the sensor detects a signal for about 41mm. The difference in the position of the laser line reflected on the sensor active area [8] in Figure 2b (Y-axis) corresponds to the height of the rubber. The real measured height of the rubber being 11.74 mm is equivalent to a height of about 3 mm (from 0 mm to 3 mm on the Y axis) measured of course on the sensor active area after a lens reduction [8]. 

The stability of the profile detected is acceptable, even though a slight flickering is observed, which could be attributed to vibrations caused by the translation table during the scanning procedures as well as to the noise of the sensor.

## 3. 3D Sensor MATLAB/SIMULINK Simulation

A MATLAB/SIMULINK model has been constructed in order to analyze and view the possibilities of the PSD array sensor technology before it is further expanded or developed.

The simulation reads any “PLY” 3D data file located inside a predefined directory. In our case, the file “teapot.ply” is used. This file contains the raw 3D cloud data of the object that is to be scanned in 3D, and, in this case, it is a teapot; however any other predefined 3D object can be employed.

When the function “ply_read.m” is executed, the simulation reads the object. Two matrices, “[Tri, Pts]”, are then recorded, one regarding the triangular connectivity information (triangle faces) and another regarding the VERTEX information (vertex or 3D points) of the object to be scanned in 3D within the simulation.

**Figure 3:**
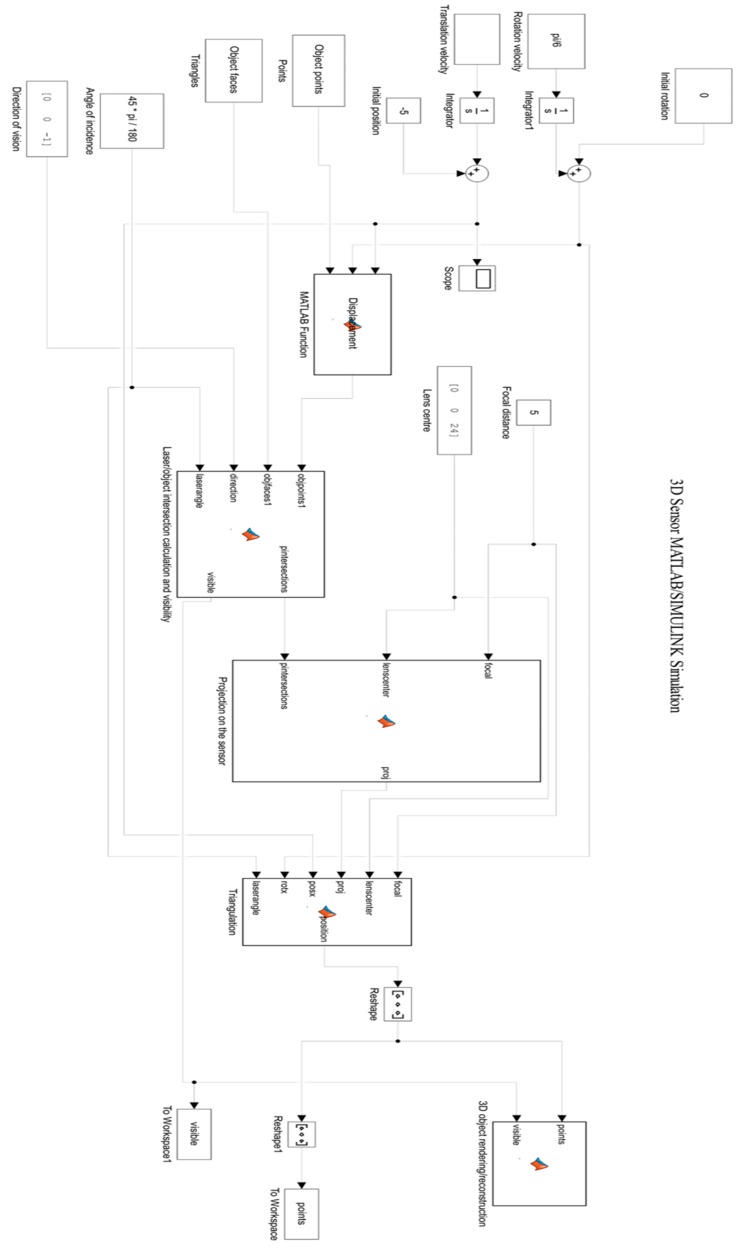
Structure of the SIMULINK simulation model with all its component blocks.

Figure 3 shows the structure of the simulation model with all its component blocks. Relevant blocks from this SIMULINK model are explained.

### 3.1. Initial Rotation

The object can be rotated before starting the scan. Any angle of initial rotation can be defined, e.g., (15°), however it should be entered/translated to radians (instead of degrees), so for 15° case, the term 15π/180 should be inserted. The default value is 0. 

### 3.2. Rotation Velocity

The velocity of rotation is defined as the speed in which the object rotates per second. For example, a value of 36° per second could be inserted since the default value of the simulation is 10 s and this would result in the object rotating 360° in 10 s. The default value is 0. The value should be inserted in radians per second, instead of degrees, so therefore it would be 36π/180 or 2π/10. If we are only interested in rotating the object without translating, we can change the velocity of rotation to 2π/10 and change the translation velocity to 0 so that the object does not move. In addition, we can set the initial position to 0 so that the object is right below the lens and a better scan would be obtained in this particular case. 

### 3.3. Translation Velocity

The translation velocity is defined as the speed in which the object translates, per second, in the X direction (X-axis). For example, a value of 1 cm/s could be inserted. The default value is 1. Thus, in order to rotate and translate the object at the same time, a value of rotation velocity of for example, 2π/10 can be inserted in combination with the default value of 1cm/s for the translation velocity and the default value of initial position of −5 cm (see Section 3.4)).

### 3.4. Initial Position

This value determines the position (generally in the x-axis) where the object starts to be scanned in relation to the 0, 0, 0 (x, y, z) point or center of the system, perpendicular to the lens and 3D sensor. The default value is −5 cm in the X-axis, which is from where the object starts to be scanned along the X-axis. The center position is at the value x = 0, right below the lens.

### 3.5. Object Points

This value refers to the “Pts” matrix of points or (x, y, z) array of points returned by the ply_read.m function.

### 3.6. Object Faces

This value refers to the “Tri” matrix of faces or triangular order for the connectivity of the points returned by the ply_read.m function.

### 3.7. Angle of Incidence

This is the viewing or scanning angle, which exists between the laser and the normal to the lens/sensor system. A default value of 45π/180 has been set (45° in radians), however it can be changed in order to scan the object at various other angles. For example, a viewing/scanning angle of 15° will yield a different result to a 45° angle, since the laser would be projected differently and on different parts/sides of the object.

### 3.8. Direction of Vision

This parameter indicates the direction in which the lens/sensor system is viewing the object. This parameter is composed of three coordinates (x, y, z). The default value is [0, 0, −1], indicating that the direction of vision is downwards in the −Z direction (negative Z-axis). In our system setup, this value is currently fixed since the object is passing and is being scanned below the lens/sensor system as it moves in the X-axis.

### 3.9. Displacement

This SIMULINK block calculates the displacement taking into account the parameters of translation and rotation velocity. The displacement is calculated as the simulation runs on every sample interval.

### 3.10. Focal Distance

This is the focal distance provided by the physical lens used in the real system setup, which, in this case, is set to a default value of 5 cm. Of course, in this simulation, it can also be changed to other values.

### 3.11. Lens Centre

This parameter defines where the center of the lens is located with reference to the 0, 0, 0 (x, y, z) point, which is located where the object meets the normal of the lens/sensor system. The default value is [0, 0, 24], so this means that the center of the lens is located 24 cm above the [0, 0, 0] reference point, so 24 cm in the Z-axis (upwards). This corresponds to the reality, since in the real system setup, the center of the lens is also located 24 cm above the [0, 0, 0] reference point.

### 3.12. Laser/Object Intersection Calculation and Visibility

This SIMULINK block considers the points of the object, the triangular object faces formed by those points from the object, the direction of vision, the viewing/scanning angle of the laser and the laser sheet of light itself in order to calculate the intersection of the object points/faces with the laser. The visibility of those intersecting points is also determined.

For a 32 PSD sensor, the physical number of detectors on the sensor is 32 and thereby the “numrays” variable defined in the code of the SIMULINK block is set to 32. This variable may be changed by accessing the code in the MATLAB workspace and by simply modifying the value of “numrays = 32” to any other value, taking into account that usually the physical number of detectors in these kind of PSD array sensors should vary from 32 through to 128, 256, 512 or 1024. The higher the number of detectors, the higher the resolution in the Y- axis.

Once the value is modified, the model should be “re-built” and thereafter updated using the icon “Build Model” on the MATLAB menu bar. The SIMULINK model should now be ready to “RUN” with the new value. 

### 3.13. Projection on the Sensor

This SIMULINK block calculates and performs the 2D projection of the scanned object points or 2D frames on the sensor active area at each sample interval as the simulation runs.

### 3.14. Triangulation

This SIMULINK block uses the triangulation formulae and its relevant parameters in order to calculate the 3D coordinates (x, y, z) of each of the scanned points from the object at each sample interval.

### 3.15. 3D Object Rendering/Reconstruction

This SIMULINK block uses the previously calculated 3D coordinates and 2D frames to reconstruct or render the object shape in 3D. The 3D object mesh of points is plotted as a 3D map at each sample interval as the simulation runs.

### 3.16. Points

The matrix or array of points is returned to the MATLAB workspace at each sample interval. 

### 3.17. Visible

The matrix or array of visible points is returned to the MATLAB workspace at each sample interval.

### 3.18. Running the Simulation

The simulation is compiled and run with the default parameters for each block; however, as already described, most of these can be modified to suit the needs of the required 3D scan.

**Figure 4:**
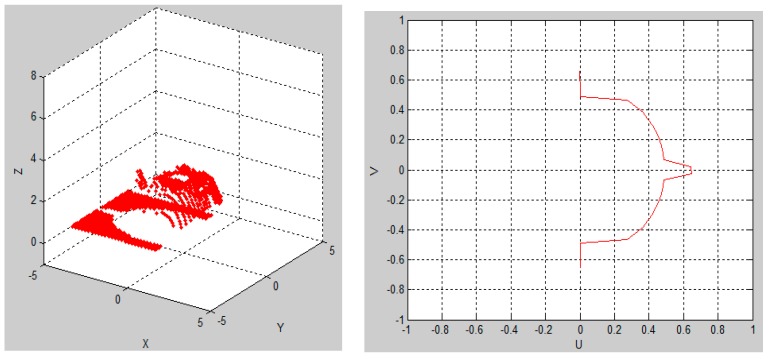
Simulation running when default parameters were used.

**Figure 5:**
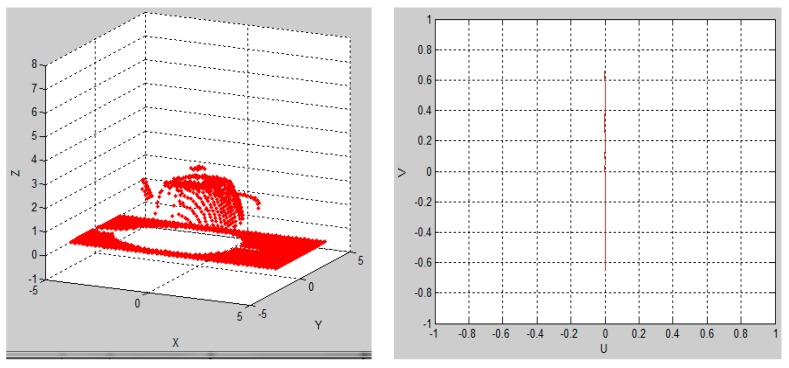
Simulation results at the end of the 3D object scan simulation.

Figure 4 shows the simulation running when the default parameters were used and Figure 5 presents the simulation results at the end of the simulation.

**Figure 6:**
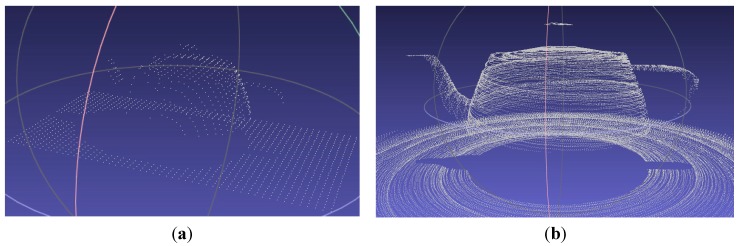
(**a**) Scanned teapot (3D point mesh) viewed in MESHLAB; (**b**) Best 3D scanning result (3D point mesh) viewed in MESHLAB, obtained with a 32 PSD sensor array.

In Figure 4 and Figure 5, the teapot was scanned and rendered using a 0.2 s sample time at a 45° scanning angle, a translation velocity of 1 cm/s, an initial position of –5 cm and no rotation velocity (0). At the end of the simulation, the relevant scanned 3D object generated data can be exported and, in that case, a file of “xyz” format called “puntos.xyz” is stored in a predefined directory. The file “puntos.xyz” can now be opened using a 3D mesh visualization program, such as MESHLAB. Figure 6a shows the generated file “puntos.xyz” opened in MESHLAB. Here, the teapot (object), which was scanned in the 3D sensor simulation, is illustrated and we can ZOOM IN, rotate and have a closer look at the resulting mesh of 3D points. Figure 6b shows the best 3D scanning results obtained when using a 32 PSD array sensor, starting at the default initial position of –5 cm, translating 1 cm/s, rotating 360° 10 times in 10 s, while using a sample time of 1/360 s.

### 3.19. Expansion of the PSD Array Size

**Figure 7:**
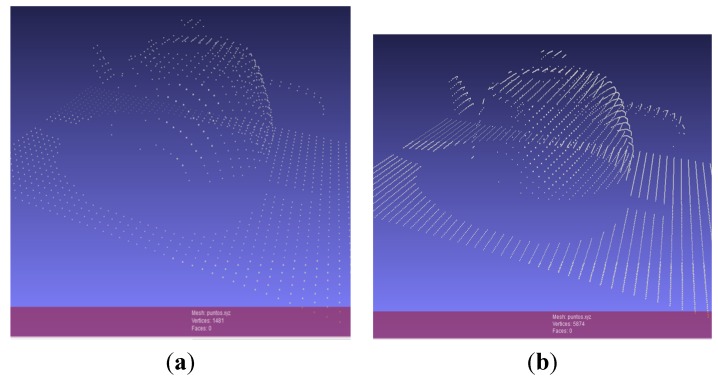

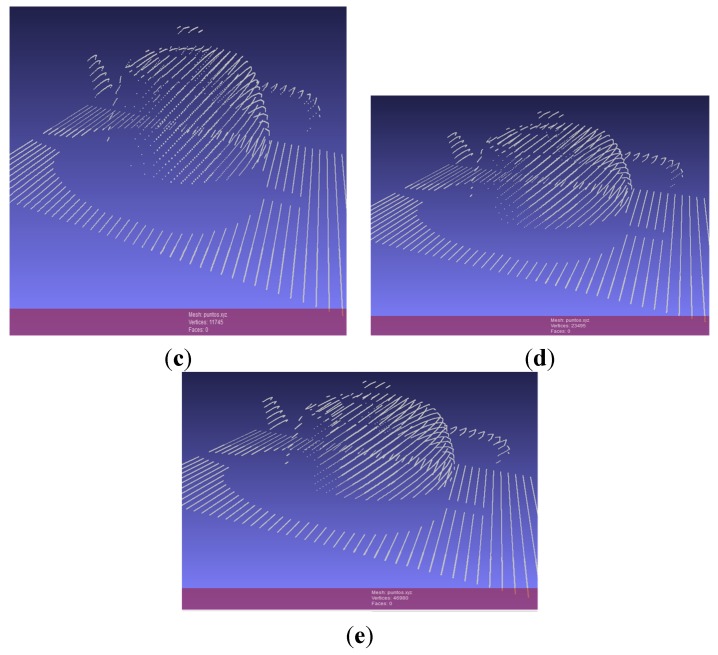
Expansion of the simulated PSD array size: (**a**) 32 PSD; (**b**) 128 PSD; (**c**) 256 PSD; (**d**) 512 PSD; and (**e**) 1024 PSD.

As referred to in Section 3.12, the default number of simulated detectors on the sensor is 32 and can be expanded to any desired value, although the proposed sizes are 128, 256, 512 and 1024. The higher the number of detectors, the higher the resolution in the Y-axis and the higher the definition and quality of the 3D scanned object. Such a fact is clearly noticeable in Figure 7a–e, where we can see how the number of points or vertices of the scanned 3D object increases as we increase the number of detectors in the PSD sensor array.

### 3.20. Object Resolution

The “teapot.ply” object file used in this simulation has 279 vertices and 500 faces. A lower resolution “teapot.ply” file does not affect the outcome of this research since the simulation calculates the point of intersection of the laser line with the surface of the object at each time frame and so it is scanning exactly what comes in the file. Even if the object’s resolution is lower or higher, the simulation will still scan the object’s surface at the intersection points between the laser line and the object. Therefore, the resolution does not depend on the number of vertices or faces and it will scan it as it is.

Since “teapot.ply” is composed of polygon faces that do not exactly represent the 3D profile of a real teapot composed of curved surfaces, a file with the highest possible resolution will of course best mimic the object being 3D scanned/simulated and provide the most precise estimate of a real teapot.

## 4. Triangulation Platform Configuration and Formulae

The triangulation configuration sketch and formulae derived by Park and DeSouza (see Figure 6.4 in reference [10]) was used to construct the simulation depicted in Figure 6 and Figure 7, which corresponds to the real physical triangulation 3D sensor system scenario, which is being simulated in this SIMULINK model.

The coordinates of each of the 3D scanned points are calculated in accordance to the properties of similar triangles.

The Z-coordinate is calculated using the following expression [10]:

(1) $$z=\frac{fb}{p+f\text{ tan }\theta }$$

where *f* is the focal length of the camera, *p* is the image coordinate of the illuminated point, *θ* is the incident/scanning angle, *Xc* and *Zc* (see Figure 6.4 in reference [10]) are two of the three principle axes of the camera coordinate system, respectively, and *b* (baseline) is the distance between the focal point and the laser along the *Xc* axis (see Figure 6.4 in reference [10]).

The X-coordinate is calculated using the following expression [10]:

(2) x=b−z tan θ

The Y-coordinate is calculated using the following expression also from the principle of similar triangles:

(3) $$y=\frac{Yq}{z}$$

where y is the distance along individual detectors on the sensor measured from the middle point of the sensor on the Y-axis on the sensor active area, Y is the distance measured from the middle point of the translation table (where object is placed) along the Y-axis within the scanning area, q is the distance between the sensor and the lens which is not the focal distance f, and z is the perpendicular distance between the lens and the object or the Z coordinate.

The error in the Z-coordinate measurement, Δz, is obtained by differentiating the equation of the Z-coordinate (Equation (1)), and the resulting expression is illustrated below [10]:

(4) $$\Delta z=\frac{z^{2}}{fb}\Delta p+\frac{z^{2}{\text{sec}}^{2}\theta }{b}\Delta \theta$$

where Δp and Δθ are the measurement errors of p and θ, respectively.

The results obtained by the physical sensor system (e.g., Figure 2b) when measuring object profiles in 3D conclude that the error of the sensor system when detecting objects in the existing triangulation platform setup is of ~5% in the X-axis and of ~5.6% in the Z-axis, which could be attributed to possible vibrations of the translation table (object) movement during the scanning process as well as to sensor noise. Signal fluctuations (noise in the signal) on the scanned profile define the object measurement error in the Z-axis and the maximum and minimum values of such fluctuations define such error interval.

## 5. Conclusions and Future Work

It has been demonstrated that a-Si:H 128 PSD sensor arrays and their corresponding systems work correctly and can be used as high speed and high resolution sheet-of-light 3D object scanning systems. The constructed simulation shows a huge potential for the proposed 3D sensor technology, which is clearly able to compete with the most advanced CMOS sensor-based sheet-of-light laser triangulation 3D cameras. Improvements are needed in order to achieve 100% correct sensor position response, especially for the 128 PSD array sensor and such a goal could be attained by fabricating a sensor using a different material, such as nanocrystalline silicon, which hardly degrades the overall position response of the structure over time. Other foreseen restrictions are expected when miniaturizing the sensor, for example to 256, 512 or 1024 elements, now that even if the technology for that purpose exists, problems may occur during material layer fabrication procedures, such as possible short circuiting in sensor channels, *etc*. Other limitations exist within the triangulation platform. Better optics and equipment can be used to improve the 3D detection setup overall. Anti-vibration and sensor noise reduction measures could be introduced, too. However, system hardware and software do not need to be enhanced.

The successful integration of amorphous silicon PSD array sensors into suitable sheet-of-light 3D object rendering systems is now possible and feasible. The quality and realism of the 3D object profiles depends on the correct and accurate position response of each detector from the sensor as well as on the size of the PSD array, meaning that the higher the number of PSDs integrated on the array, the higher the 3D object profile resolution on the discrete sensor axis, and, subsequently, the higher the number of total image 3D scan points.
